# Rates of and Factors Associated With Primary and Booster COVID-19 Vaccine Receipt by US Veterans, December 2020 to June 2022

**DOI:** 10.1001/jamanetworkopen.2022.54387

**Published:** 2023-02-02

**Authors:** Kristina L. Bajema, Mazhgan Rowneki, Kristin Berry, Amy Bohnert, C. Barrett Bowling, Edward J. Boyko, Theodore J. Iwashyna, Matthew L. Maciejewski, Ann M. O’Hare, Thomas F. Osborne, Elizabeth M. Viglianti, Denise M. Hynes, George N. Ioannou

**Affiliations:** 1 Veterans Affairs Portland Health Care System, Portland, Oregon; 2 Division of Infectious Diseases, Department of Medicine, Oregon Health and Science University, Portland; 3 Center of Innovation to Improve Veteran Involvement in Care, Veterans Affairs Portland Health Care System, Portland, Oregon; 4 Center of Innovation for Veteran Centered Value Driven Care, Veterans Affairs Puget Sound Healthcare System, Seattle, Washington; 5 Center for Clinical Management Research, Veterans Affairs Ann Arbor Health Care System, Ann Arbor, Michigan; 6 Department of Anesthesiology, University of Michigan, Ann Arbor; 7 Durham Veterans Affairs Geriatric Research Education and Clinical Center, Durham Veterans Affairs Medical Center, Durham, North Carolina; 8 Department of Medicine, Duke University, Durham, North Carolina; 9 Seattle Epidemiologic Research and Information Center, Veterans Affairs Puget Sound Healthcare Seattle, Washington; 10 Center for Clinical Management Research, Veterans Affairs Ann Arbor Healthcare System, Ann Arbor, Michigan; 11 Schools of Medicine and Public Health, Johns Hopkins, Baltimore, Maryland; 12 Department of Population Health Sciences, Duke University School of Medicine, Durham, North Carolina; 13 Duke-Margolis Center for Health Policy, Duke University, Durham, North Carolina; 14 Center of Innovation to Accelerate Discovery and Practice Transformation, Durham Veterans Affairs Medical Center, Durham, North Carolina; 15 Veterans Affairs Puget Sound Healthcare Seattle, Washington; 16 Department of Medicine, University of Washington, Seattle; 17 Veterans Affairs Palo Alto Healthcare System, Palo Alto, California; 18 Department of Radiology, Stanford University School of Medicine, Stanford, California; 19 Department of Internal Medicine, University of Michigan Medical School, Ann Arbor; 20 Health Management and Policy, School of Social and Behavioral Health Sciences, College of Public Health and Human Sciences, Health Data and Informatics Program, Center for Quantitative Life Sciences, Oregon State University, Corvallis; 21 Division of Gastroenterology, Veterans Affairs Puget Sound Health Care System and University of Washington, Seattle; 22 Research and Development, Veterans Affairs Puget Sound Health Care System, Seattle, Washington

## Abstract

**Importance:**

COVID-19 vaccination rates remain suboptimal in the US. Identifying factors associated with vaccination can highlight existing gaps and guide targeted interventions to improve vaccination access and uptake.

**Objective:**

To describe incidence and patient characteristics associated with primary, first booster, and second booster COVID-19 vaccination in the Veterans Health Administration (VHA).

**Design, Setting, and Participants:**

This retrospective cohort study assessed US veterans receiving care in VHA medical centers and outpatient clinics as of December 1, 2020. All VHA enrollees with an inpatient, outpatient, or telehealth encounter in VHA as well as a primary care physician appointment in the preceding 24 months were included.

**Exposures:**

Demographic characteristics, place of residence, prior SARS-CoV-2 infection, and underlying medical conditions.

**Main Outcomes and Measures:**

Cumulative incidence of primary, first booster, and second booster COVID-19 vaccination through June 2022. Cox proportional hazards regression was used to identify factors independently associated with COVID-19 vaccination.

**Results:**

Among 5 632 413 veterans included in the study, 5 094 392 (90.4%) were male, the median (IQR) age was 66 (51-74) years, 1 032 334 (18.3%) were Black, 448 714 (8.0%) were Hispanic, and 4 202 173 (74.6%) were White. Through June 2022, cumulative incidences were 69.0% for primary vaccination, 42.9% for first booster, and 9.3% for second booster. Cumulative incidence for primary vaccination increased with increasing age, from 46.9% (95% CI, 46.8%-47.0%) among veterans aged 18 to 49 years to 82.9% (95% CI, 82.8%-83.0%) among veterans aged 80 to 84 years. More Black veterans completed primary vaccination (71.7%; 95% CI, 71.6%-71.8%) compared with White veterans (68.9%; 95% CI, 68.9%-69.0%), and more urban-dwelling veterans completed primary vaccination (70.9%; 95% CI, 70.9%-71.0%) compared with highly rural-dwelling veterans (63.8%; 95% CI, 63.4%-64.1%). Factors independently associated with higher likelihood of both primary and booster vaccination included older age, female sex, Asian or Black race, Hispanic ethnicity, urban residence, and lack of prior SARS-CoV-2 infection.

**Conclusions and Relevance:**

In this cohort study of US veterans, COVID-19 vaccination coverage through June 2022 was suboptimal. Primary vaccination can be improved among younger, rural-dwelling veterans. Greater uptake of booster vaccination among all veterans is needed.

## Key Points

**Question:**

What was the uptake of and factors associated with COVID-19 primary and booster vaccination in the Veterans Health Administration from December 2020 to June 2022?

**Findings:**

In this cohort study of 5 632 413 enrolled veterans, cumulative incidences were 69.0% for primary vaccination, 42.9% for first booster, and 9.3% for second booster. Older age, Asian or Black race, Hispanic ethnicity, and urban residence were independently associated with receipt of vaccination.

**Meaning:**

These findings suggest targeted outreach to younger, rural veterans may improve COVID-19 vaccination rates.

## Teaser

This cohort study examines factors associated with receipt of COVID-19 vaccination and rates of vaccination among US veterans.

## Introduction

COVID-19 vaccines are highly effective in preventing severe COVID-19 illness and death and have been recommended for everyone 6 months or older in the US.^1,2^ To date, 4 COVID-19 vaccines have been approved or authorized under US Food and Drug Administration (FDA) Emergency Use Authorization (EUA). The FDA EUA was first issued for the BNT162b2 (Pfizer-BioNTech) and mRNA-1273 (Moderna) COVID-19 messenger RNA (mRNA) vaccines in December 2020 followed by JNJ-78436735 (Janssen/Johnson & Johnson) in February 2021 and NVX-CoV2373 (Novavax) in July 2022 (eTable 1 in Supplement 1).^3^ In September 2021, the FDA authorized a booster dose of the Pfizer-BioNTech COVID-19 vaccine in certain populations, with subsequent booster authorization for Moderna and Janssen products in October 2021. Second booster vaccination, first authorized by the FDA in March 2022, was recommended for all adults 50 years or older as well as for persons 12 years or older who were moderately or severely immunocompromised.^1,4^ After FDA authorization for bivalent formulations of the mRNA vaccines in August 2022, all persons 5 years or older are now recommended to receive bivalent booster vaccination.^5^

Despite national efforts to encourage COVID-19 vaccination, it is estimated that only approximately 77% of adults in the US had completed primary vaccination by July 2022, of whom only 51% had received a first booster dose.^6^ Few studies have comprehensively described sociodemographic, geographic, and clinical factors associated with receipt of primary and booster vaccination.^7^ The Veterans Health Administration (VHA), run by the US Department of Veterans Affairs (VA), provides comprehensive care to more than 9 million enrolled veterans in the US and worked closely with the Centers for Disease Control and Prevention and other federal partners to quickly deliver COVID-19 vaccines to veterans following initial EUA.^8^ The VHA affords an opportunity to evaluate vaccine uptake in different patient groups and promote equitable access to preventive care. We sought to describe incidence of and factors associated with receipt of COVID-19 primary, first booster, and second booster vaccination among VHA enrollees from December 2020 through June 2022.

## Methods

### Study Setting and Data Sources

The VHA is the largest integrated health care system in the US, provides care at 171 medical centers and 1113 outpatient clinics throughout the country, and uses a comprehensive nationwide electronic health record (EHR) system.^8^ We used VA’s Corporate Data Warehouse (CDW), a relational database of VHA enrollees’ EHR data.^9^ The CDW includes the COVID-19 Shared Data Resource (CSDR), supported by the VA Informatics and Computing Infrastructure, and contains information on all VHA enrollees tested for or vaccinated against COVID-19.^10^ In addition to vaccines administered through VHA, CSDR captures some COVID-19 vaccines given outside VHA (eg, pharmacies, health departments, mass vaccination centers, and clinics) and electronically reported to VHA or documented by VHA practitioners. To improve ascertainment of vaccination records, information on COVID-19 vaccination was supplemented with the Centers for Medicare & Medicaid Services (CMS) Medicare data for vaccination administered through Medicare services, as well as data from the VA’s Community Care program, which coordinates and reimburses local care provided outside VHA and is accessed through the Patient Integrity Tool.^11^ For this study, CDW and Patient Integrity Tool data were updated through June 30, 2022, and the CMS Medicare data through December 31, 2021, which represented the most recent date of available CMS Medicare data at the time of analysis. This study was approved by the VA Puget Sound Institutional Review Board, which determined that patient consent was not required. The study followed the Strengthening the Reporting of Observational Studies in Epidemiology (STROBE) reporting guideline.

### Study Population

We identified a cohort of veterans 18 years or older enrolled in VHA and alive as of December 1, 2020. We limited the study population to VHA enrollees with an inpatient, outpatient, or telehealth encounter in VHA as well as a primary care physician appointment in the preceding 24 months.

### Baseline Characteristics

We ascertained baseline demographic, geographic, and clinical characteristics documented in the 2-year period before the date of cohort entry on December 1, 2020. Race and ethnicity (associated with COVID-19 vaccination) were determined as reported in VHA EHR and enrollment records; other race included self-identification as other or more than 1 race. The latest zip code from the baseline period was used to determine Veterans Integrated Services Networks (VISNs) and rurality of residence based on the Rural-Urban Commuting Areas system.^12,13,14^ Body mass index (BMI) was calculated as weight in kilograms divided by height in meters squared using most recently measured weight and height. We determined the presence of 10 underlying conditions associated with adverse COVID-19–related outcomes (Table) based on *International Statistical Classification of Diseases and Related Health Problems, Tenth Revision* (*ICD-10*) codes recorded in the VHA EHR and on VA Community Care service claims.^15,16^ The *ICD-10* codes were also used to calculate the Charlson Comorbidity Index (CCI).^17,18^ We also determined receipt of immunosuppressive medications or cancer therapies within 2 years before cohort entry.^19^ To estimate baseline health care use, we ascertained the number of primary care, mental health, and specialty outpatient encounters for each veteran during the 2-year period before cohort entry.^20^

**Table.** Cumulative Incidence of COVID-19 Vaccination Among VA Enrollees and aHRs by Demographic, Geographic, and Clinical Factors, December 2020 to June 2022^a^^,^^b^

Characteristic	Veterans in care at the VA, No. (%)	Primary vaccination			First booster			Second booster		
		No. of events	Cumulative incidence, % (95% CI)^c^	aHR (95% CI) (n = 5 632 413)	No. of events	Cumulative incidence, % (95% CI)^c^	aHR (95% CI) (n = 3 647 739)^d^	No. of events	Cumulative incidence, % (95% CI)^c^	aHR (95% CI) (n = 1 937 404)^e^
Overall	5 632 413 (100)	3 826 436	69.0 (69.0-69.1)		2 320 475	42.9 (42.8-42.9)		497 206	9.3 (9.3-9.4)	
Sex										
Female	538 021 (9.6)	340 799	63.6 (63.5-63.7)	1.07 (1.06-1.07)	180 516	33.9 (33.8-34.0)	1.09 (1.08-1.09)	34 077	6.4 (6.4-6.5)	1.10 (1.09-1.12)
Male	5 094 392 (90.4)	3 485 637	69.6 (69.6-69.7)	1 [Reference]	2 139 959	43.8 (43.8-43.9)	1 [Reference]	463 129	9.6 (9.6-9.7)	1 [Reference]
Age group, y										
18-49	1 327 019 (23.6)	621 623	46.9 (46.8-47.0)	1 [Reference]	208 205	15.7 (15.7-15.8)	1 [Reference]	11 817	0.9 (0.9-0.9)	1 [Reference]^f^
50-59	807 261 (14.3)	517 004	64.3 (64.2-64.4)	1.54 (1.53-1.54)	269 148	33.7 (33.6-33.8)	1.77 (1.76-1.79)	47 215	5.9 (5.9-6.0)	1.80 (1.72-1.89)
60-69	1 109 186 (19.7)	802 440	73.2 (73.1-73.3)	2.03 (2.02-2.04)	513 886	47.7 (47.6-47.8)	2.59 (2.57-2.6)	121 161	11.4 (11.3-11.4)	2.57 (2.45-2.70)
70-74	1 065 086 (18.9)	836 432	79.8 (79.7-79.9)	2.91 (2.90-2.92)	592 841	58.0 (57.9-58.1)	3.47 (3.45-3.49)	146 324	14.6 (14.5-14.6)	2.85 (2.71-2.98)
75-79	598 366 (10.6)	483 263	82.6 (82.5-82.7)	3.68 (3.67-3.70)	350 411	62.0 (61.9-62.2)	3.73 (3.71-3.75)	87 449	15.8 (15.8-16.0)	2.97 (2.83-3.12)
80-84	307 600 (5.5)	246 221	82.9 (82.8-83.0)	3.94 (3.92-3.96)	175 559	62.4 (62.3-62.6)	3.68 (3.65-3.70)	40 326	14.9 (14.7-15.0)	2.75 (2.62-2.88)
85-89	249 994 (4.4)	196 194	82.8 (82.6-83.0)	4.19 (4.17-4.22)	134 125	61.8 (61.6-62.0)	3.40 (3.37-3.43)	28 206	13.7 (13.6-13.9)	2.42 (2.30-2.54)
≥90	167 901 (3.0)	123 259	80.8 (80.6-81.0)	4.14 (4.12-4.17)	76 300	58.8 (58.5-59.1)	2.87 (2.85-2.9)	14 708	12.5 (12.4-12.7)	2.15 (2.04-2.27)
Race										
American Indian or Alaska Native	64 574 (1.1)	39 720	62.4 (62.1-62.8)	0.95 (0.94-0.96)	21 827	35.0 (34.6-35.4)	0.96 (0.95-0.98)	4285	7.0 (6.8-7.2)	0.98 (0.95-1.02)
Asian	80 031 (1.4)	60 113	75.7 (75.4-76.0)	1.48 (1.46-1.49)	36 181	46.0 (45.7-46.4)	1.36 (1.34-1.37)	8403	10.8 (10.5-11.0)	1.47 (1.44-1.51)
Black	1 032 334 (18.3)	730 522	71.7 (71.6-71.8)	1.10 (1.11-1.12)	448 133	44.7 (44.6-44.8)	1.15 (1.14-1.15)	101 301	10.2 (10.1-10.3)	1.24 (1.23-1.25)
Native Hawaiian or Other Pacific Islander	58 643 (1.0)	40 405	69.9 (69.5-70.3)	1.08 (1.07-1.09)	23 864	42.1 (41.7-42.5)	1.04 (1.03-1.06)	5176	9.2 (9.0-9.5)	1.05 (1.02-1.09)
White	4 202 173 (74.6)	2 846 576	68.9 (68.9-69.0)	1 [Reference]	1 741 626	43.3 (43.-43.4)	1 [Reference]	370 444	9.4 (9.3-9.4)	1 [Reference]
Other^g^	5900 (0.1)	4701	81.2 (80.2-82.2)	1.10 (1.07-1.13)	3331	59.1 (57.8-60.4)	1.11 (1.07-1.15)	880	15.9 (14.9-16.8)	1.08 (1.00-1.17)
Missing	188 758 (3.4)	104 399	55.6 (55.3-55.8)	1 (0.99-1.00)	45 513	24.3 (24.2-24.5)	0.94 (0.93-0.95)	6717	3.6 (3.5-3.7)	1.08 (1.05-1.11)
Ethnicity										
Hispanic or Latino	448 714 (8.0)	313 940	70.8 (70.7-70.9)	1.11 (1.11-1.12)	186 069	42.6 (42.5-42.8)	1.07 (1.06-1.08)	436 669	10.2 (10.1-10.2)	1.11 (1.09-1.12)
Not Hispanic or Latino	4 946 545 (87.8)	3 360 312	69.1 (69.0-69.1)	1 [Reference]	2 047 254	43.1 (43.1-43.2)	1 [Reference]	43 900	9.3 (9.3-9.4)	1 [Reference]
Missing	237 154 (4.2)	152 184	65.3 (65.1-65.5)	0.99 (0.98-0.99)	87 152	38.3 (38.1-38.5)	1.00 (1.00-1.01)	16 637	7.4 (7.3-7.5)	0.95 (0.93-0.96)
Residence^h^										
Urban	3 771 263 (67.0)	2 632 787	70.9 (70.9-71.0)	1 [Reference]	1 618 052	44.6 (44.5-44.6)	1 [Reference]	363 448	10.2 (10.1-10.2)	1 [Reference]
Rural	1 769 184 (31.4)	1 135 976	65.3 (65.2-65.4)	0.81 (0.81-0.81)	668 246	39.4 (39.4-39.5)	0.84 (0.83-0.84)	126 919	7.6 (7.6-7.6)	0.78 (0.78-0.79)
Highly rural	79 790 (1.4)	49 975	63.8 (63.4-64.1)	0.70 (0.70-0.71)	29 386	38.6 (38.2-38.9)	0.73 (0.72-0.74)	5786	7.7 (7.5-7.9)	0.75 (0.72-0.77)
Missing	12 176 (0.2)	7698	64.3 (63.4-65.2)	0.57 (0.56-0.59)	4791	40.7 (39.8-41.6)	0.77 (0.75-0.80)	1053	9.0 (8.5-9.5)	0.82 (0.76-0.88)
VISN and region										
Midwest	1 101 919 (19.6)	758 839	70.0 (69.9-70.1)	1.10 (1.10-1.11)	501 227	47.5 (47.4-47.6)	1.28 (1.28-1.29)	115 189	11.1 (11.1-11.2)	1.43 (1.42-1.45)
10	411 513 (7.3)	269 568	66.7 (66.5-66.8)	0.96 (0.95-0.96)	172 339	43.8 (43.7-44.0)	1.10 (1.09-1.10)	37 418	9.7 (9.6-9.8)	1.10 (1.08-1.11)
12	280 258 (5.0)	201 807	73.2 (73.1-73.4)	1.14 (1.13-1.14)	138 029	51.5 (51.3-51.6)	1.25 (1.25-1.26)	34 893	13.2 (13.1-13.4)	1.44 (1.42-1.46)
15	172 500 (3.06)	110 591	65.2 (65.0-65.4)	0.99 (0.99-1.00)	67 604	40.9 (40.7-41.2)	1.09 (1.08-1.1)	12 489	7.7 (7.6-7.8)	0.98 (0.96-1.01)
23	237 648 (4.2)	176 873	75.5 (75.3-75.7)	1.36 (1.36-1.37)	123 255	54.0(53.8-54.2)	1.48 (1.47-1.49)	30 389	13.6 (13.4-13.7)	1.59 (1.57-1.62)
Northeast	902 660 (16.0)	648 251	73.1 (73.0-73.2)	1.14 (1.13-1.14)	420 880	48.7 (48.6-48.8)	1.22 (1.22-1.23)	94 286	11.1 (11.0-11.2)	1.31 (1.31-1.33)
1	240 609 (4.3)	178 053	75.2 (75.1-75.4)	1.20 (1.20-1.21)	119 125	51.7 (51.5-51.9)	1.25 (1.24-1.26)	29 430	13.0 (12.8-13.1)	1.39 (1.36-1.41)
2	176 961 (3.1)	130 977	75.4 (75.2-75.6)	1.15 (1.14-1.16)	87 241	51.6 (51.3-51.8)	1.18 (1.17-1.19)	20 547	12.4 (12.2-12.5)	1.21 (1.19-1.23)
4	293 900 (5.2)	210 800	73.2 (73.1-73.4)	1.09 (1.09-1.10)	140 448	50.2 (50.0-50.4)	1.18 (1.17-1.18)	29 832	10.9 (10.8-11.0)	1.11 (1.09-1.13)
5	191 190 (3.4)	128 421	68.2 (68.0-68.4)	1.02 (1.01-1.03)	74 066	40.2 (40.0-40.4)	0.98 (0.97-0.99)	14 477	8.0 (7.8-8.1)	0.95 (0.93-0.97)
South	2 291 225 (40.7)	1 506 941	66.8 (66.8-66.9)	1	860 039	39.0 (38.9-39.0)	1	163 804	7.5 (7.5-7.6)	1
6	399 083 (7.1)	264 107	67.2 (67.0-67.3)	1.00 (0.99-1.00)	157 283	40.8 (40.7-41.0)	1.01 (1.00-1.01)	31 471	8.3 (8.2-8.4)	0.95 (0.93-0.96)
7	418 629 (7.4)	278 811	67.7 (67.5-67.8)	1.00 (1.00-1.01)	149 007	36.9 (36.7-37.0)	0.85 (0.85-0.86)	23 909	6.0 (5.9-6.1)	0.70 (0.69-0.71)
8	472 938 (8.4)	325 175	69.8 (69.7-70.0)	1 [Reference]	198 899	43.8 (43.6-43.9)	1 [Reference]	41 902	9.4 (9.3-9.4)	1 [Reference]
9	247 365 (4.4)	154 251	63.4 (63.3-63.6)	0.92 (0.92-0.93)	89 271	37.6 (37.4-37.8)	0.97 (0.96-0.98)	15 245	6.5 (6.4-6.6)	0.79 (0.77-0.81)
16	367 448 (6.5)	231 524	64.1 (63.9-64.2)	0.93 (0.93-0.94)	130 995	37.2 (37.0-37.3)	0.92 (0.92-0.93)	26 049	7.5 (7.4-7.6)	0.95 (0.94-0.97)
17	385 762 (6.9)	253 073	66.5 (66.4-66.7)	1.01 (1.01-1.02)	134 584	36.0 (35.8-36.2)	0.91 (0.90-0.91)	25 228	6.8 (6.7-6.9)	0.89 (0.87-0.90)
West	1 276 997 (22.7)	863 250	68.6 (68.5-68.7)	1.04 (1.04-1.04)	501 960	40.8 (40.7-40.9)	1.04 (1.04-1.05)	112 384	9.3 (9.2-9.3)	1.36 (1.35-1.38)
19	296 814 (5.3)	185 415	63.4 (63.2-63.6)	0.98 (0.97-0.98)	103 511	36.2 (36.0-36.4)	0.98 (0.97-0.98)	21 446	7.6 (7.5-7.7)	1.13 (1.11-1.15)
20	280 117 (5.0)	181 542	65.6 (65.4-65.8)	0.96 (0.95-0.96)	95 271	35.2 (35.1-35.4)	0.85 (0.84-0.86)	17 081	6.4 (6.3-6.5)	0.97 (0.95-0.99)
21	280 148 (5.0)	197 679	71.7 (71.5-71.8)	1.05 (1.04-1.05)	124 631	46.3 (46.1-46.5)	1.06 (1.06-1.07)	31 736	12.0 (11.8-12.1)	1.40 (1.38-1.43)
22	419 918 (7.5)	298 614	72.3 (72.1-72.4)	1.10 (1.09-1.10)	178 547	44.1 (43.9-44.2)	1.04 (1.04-1.05)	42 121	10.5 (10.4-10.6)	1.31 (1.29-1.33)
Missing VISN	59 612 (1.1)	49 155	83.8 (83.5-84.1)	1.11 (1.10-1.12)	36 369	63.5 (63.1-63.9)	1.18 (1.16-1.19)	11 543	20.4 (20.1-20.8)	1.61 (1.57-1.65)
Prior SARS-CoV-2 infection^i^										
Yes	663 703 (11.8)	431 335	67.1 (67.0-67.2)	0.37 (0.37-0.38)	243 762	39.2 (39.0-39.3)	0.53 (0.53-0.53)	50 560	8.4 (8.3-8.4)	0.73 (0.73-0.74)
No	4 968 710 (88.2)	3 395 101	69.3 (69.3-69.3)	1 [Reference]	2 076 713	43.3 (43.3-43.4)	1 [Reference]	446 646	9.4 (9.4-9.5)	1 [Reference]
BMI										
<18.5	47 330 (0.8)	27 986	63.9 (63.5-64.4)	0.76 (0.75-0.77)	14 564	37.0 (36.5-37.4)	0.72 (0.71-0.73)	2926	7.9 (7.6-8.2)	0.83 (0.80-0.87)
18.5 to <25	1 015 185 (18.0)	662 811	67.1 (67.1-67.2)	1 [Reference]	394 528	41.6 (41.5-41.7)	1 [Reference]	84 276	9.1 (9.1-9.2)	1 [Reference]
Overweight	1 982 640 (35.2)	1 366 350	69.9 (69.9-70.0)	1.15 (1.15-1.16)	849 246	44.4 (44.3-44.5)	1.13 (1.13-1.13)	184 691	9.8 (9.8-9.8)	1.05 (1.04-1.06)
Obese I	1 469 359 (26.1)	1 017 008	70.0 (69.9-70.1)	1.20 (1.20-1.21)	622 708	43.6 (43.5-43.6)	1.16 (1.15-1.17)	134 017	9.5 (9.4-9.5)	1.04 (1.03-1.05)
Obese II	646 630 (11.5)	447 579	70.0 (69.9-70.1)	1.22 (1.21-1.22)	269 100	42.8 (42.6-42.9)	1.16 (1.15-1.17)	57 785	9.3 (9.2-9.4)	1.04 (1.03-1.05)
Obese III	329 365 (5.9)	226 983	69.8 (69.7-70.0)	1.19 (1.18-1.19)	133 192	41.8 (41.6-41.9)	1.13 (1.12-1.13)	28 394	9.0 (8.9-9.1)	1.02 (1.00-1.03)
Missing	141 904 (2.5)	77 719	55.6 (55.3-55.9)	1.08 (1.07-1.08)	37 137	27.1 (26.8-27.3)	1.05 (1.04-1.06)	5117	3.8 (3.7-3.9)	1.02 (0.99-1.06)
CCI										
0	2 789 879 (49.5)	1 688 972	60.9 (60.8-61.0)	1 [Reference]	915 219	33.3 (33.2-33.4)	1 [Reference]	161 466	5.9 (5.9-5.9)	1 [Reference]
1	1 143 086 (20.3)	821 942	73.0 (72.9-73.1)	1.06 (1.06-1.06)	514 588	46.7 (46.6-46.8)	1.00 (1.00-1.00)	109 109	10.1 (10.0-10.1)	1.00 (0.99-1.01)
2	754 978 (13.4)	576 861	78.0 (77.9-78.1)	1.08 (1.08-1.08)	386 492	53.9 (53.8-54.0)	0.99 (0.99-1.00)	91 813	13.2 (13.0-13.2)	1.01 (1.00-1.02)
3	376 787 (6.7)	294 154	80.5 (80.4-80.7)	1.08 (1.07-1.08)	200 266	57.5 (57.3-57.6)	0.95 (0.94-0.95)	50 924	15.1 (15.0-15.2)	1 (0.98-1.01)
4	238 746 (4.2)	189 173	82.3 (82.1-82.5)	1.09 (1.08-1.09)	130 662	60.2 (60.0-60.4)	0.93 (0.92-0.93)	34 254	16.4 (16.2-16.6)	0.98 (0.97-0.99)
≥5	328 937 (5.8)	255 334	83.0 (82.9-83.2)	1.09 (1.08-1.09)	173 248	61.9 (61.8-62.1)	0.85 (0.85-0.86)	49 640	18.8 (18.7-19.0)	0.98 (0.98-1.00)
Chronic kidney disease										
No	5 068 252 (90.0)	3 388 889	67.7 (67.7-67.8)	1 [Reference]	2 025 245	41.3 (41.2-41.3)	1 [Reference]	422 803	8.7 (8.7-8.8)	1 [Reference]
Yes	564 161 (10.0)	437 547	81.1 (81.0-81.2)	1.02 (1.02-1.03)	295 230	58.5 (58.3-58.6)	0.93 (0.93-0.93)	74 403	15.4 (15.3-15.5)	0.96 (0.96-0.97)
COPD										
No	4 918 208 (87.3)	3 293 278	67.8 (67.8-67.9)	1 [Reference]	1 972 590	41.4 (41.4-41.4)	1 [Reference]	411 103	8.7 (8.7-8.8)	1 [Reference]
Yes	714 205 (12.7)	533 158	77.6 (77.4-77.6)	0.98 (0.98-0.98)	347 885	53.6 (53.5-53.7)	0.90 (0.90-0.91)	86 103	13.8 (13.7-13.9)	0.98 (0.97-0.98)
Congestive heart failure										
No	5 387 941 (95.7)	3 642 782	68.6 (68.5-68.6)	1 [Reference]	2 201 168	42.3 (42.2-42.3)	1 [Reference]	465 301	9.1 (9.0-9.1)	1 [Reference]
Yes	244 472 (4.3)	183 654	80.2 (80.0-80.3)	0.93 (0.92-0.93)	119 307	57.3 (57.1-57.5)	0.83 (0.83-0.84)	31 905	16.3 (16.1-16.4)	0.95 (0.94-0.96)
Diabetes										
No	4 111 824 (73.0)	2 652 405	65.3 (65.3-65.4)	1 [Reference]	1 531 972	38.4 (38.4-38.5)	1 [Reference]	304 523	7.7 (7.7-7.8)	1 [Reference]
Yes	1 520 589 (27.0)	1 174 031	79.2 (79.1-79.3)	1.06 (1.06-1.06)	788 503	55.2 (55.1-55.3)	1.00 (1.00-1.00)	192 683	13.8 (13.8-13.9)	1.00 (0.99-1.01)
Obstructive sleep apnea										
No	4 327 550 (76.8)	2 877 786	67.6 (67.6-67.7)	1 [Reference]	1 726 508	41.6 (41.6-41.67	1 [Reference]	355 240	8.7 (8.7-8.7)	1 [Reference]
Yes	1 304 863 (23.2)	948 650	73.6 (73.6-73.7)	1.15 (1.15-1.16)	593 967	47.0 (46.9-47.0)	1.11 (1.11-1.11)	141 966	11.4 (11.3-11.4)	1.07 (1.06-1.08)
Peripheral arterial disease										
No	5 277 070 (93.7)	3 553 728	68.3 (68.3-68.4)	1 [Reference]	2 137 565	41.9 (41.9-42.0)	1 [Reference]	448 509	8.9 (8.9-8.9)	1 [Reference]
Yes	355 343 (6.3)	272 708	80.3 (80.1-80.4)	1.01 (1.01-1.02)	182 910	57.6 (57.4-57.8)	1.02 (1.02-1.03)	48 697	16.0 (15.9-16.2)	1.01 (1-1.02)
Venous thromboembolism										
No	5 543 831 (98.4)	3 761 526	68.9 (68.9-69.0)	1 [Reference]	2 277 722	42.7 (42.7-42.8)	1 [Reference]	485 695	9.2 (9.2-9.3)	1 [Reference]
Yes	88 582 (1.6)	64 910	76.5 (76.2-76.8)	0.95 (0.94-0.95)	42 753	53.3 (53.0-53.7)	0.93 (0.92-0.94)	11 511	14.9 (14.6-15.1)	0.98 (0.96-1.00)
Bipolar disorder or schizophrenia										
No	5 393 119 (95.8)	3 669 964	69.2 (69.1-69.2)	1 [Reference]	2 231 558	43.1 (43.0-43.1)	1 [Reference]	477 010	9.3 (9.3-9.4)	1 [Reference]
Yes	239 294 (4.6)	156 472	66.5 (66.3-66.6)	0.95 (0.94-0.95)	88 917	38.6 (38.4-38.8)	0.96 (0.95-0.96)	20 196	8.9 (8.8-9.0)	1.01 (1.00-1.03)
Major depressive disorder										
No	4 224 815 (75.0)	2 894 245	69.6 (69.6-69.7)	1 [Reference]	1 787 368	44.1 (44.0-44.1)	1 [Reference]	380 116	9.5 (9.5-9.6)	1 [Reference]
Yes	1 407 598 (25.0)	932 191	67.2 (67.1-67.3)	1.01 (1.01-1.02)	533 107	39.2 (39.1-39.3)	0.98 (0.97-0.98)	117 090	8.7 (8.7-8.8)	0.95 (0.95-0.96)
PTSD										
No	4 492 979 (79.8)	3 094 092	70.1 (70.0-70.1)	1 [Reference]	1 907 042	44.3 (44.3-44.4)	1 [Reference]	406 570	9.6 (9.6-9.6)	1 [Reference]
Yes	1 139 434 (20.2)	732 344	64.9 (64.8-65.0)	1.01 (1.01-1.02)	413 433	37.1 (37.0-37.2)	1.02 (1.02-1.03)	90 636	8.2 (8.2-8.3)	1.01 (1.00-1.02)
No. of primary care visits in prior 2 y										
1-5	2 237 618 (39.7)	1 369 267	62.0 (62.0-62.1)	1 [Reference]	770 084	35.6 (35.5-35.6)	1 [Reference]	123 562	5.8 (5.7-5.8)	1 [Reference]
6-11	1 645 868 (29.2)	1 150 906	70.8 (70.8-70.9)	1.11 (1.10-1.11)	701 331	44.0 (43.9-44.1)	1.04 (1.03-1.04)	149 932	9.5 (9.5-9.6)	1.23 (1.21-1.24)
≥12	1 748 927 (31.1)	1 306 263	76.4 (76.3-76.4)	1.10 (1.10-1.11)	849 060	51.3 (51.2-51.4)	1.02 (1.02-1.03)	223 712	13.8 (13.7-13.8)	1.27 (1.25-1.29)
No. of mental health visits in prior 2 y										
0	3 686 303 (65.5)	2 559 581	70.7 (70.6-70.7)	1 [Reference]	1 599 327	45.3 (45.3-45.4)	1 [Reference]	334 926	9.6 (9.6-9.7)	1 [Reference]
1-6	971 390 (17.3)	615 048	64.3 (64.2-64.4)	0.94 (0.93-0.94)	343 520	36.7 (36.6-36.8)	0.93 (0.92-0.93)	75 149	8.1 (8.1-8.2)	1.00 (0.99-1.01)
7-19	588 785 (10.5)	391 919	67.3 (67.2-67.4)	1.01 (1.01-1.01)	225 537	39.3 (39.2-39.4)	0.99 (0.98-0.99)	51 017	9.0 (8.9-9.1)	1.05 (1.04-1.06)
≥20	385 935 (6.9)	259 888	68.1 (68.0-68.3)	1.01 (1.00-1.01)	152 091	40.5 (40.4-40.7)	1.00 (1.00-1.01)	36 114	9.7 (9.6-9.8)	1.11 (1.09-1.12)
No. of specialty care visits in prior 2 y										
0	292 113 (5.2)	149 714	52.0 (51.9-52.2)	1 [Reference]	75 881	26.9 (26.8-27.1)	1 [Reference]	138 739	3.4 (3.4-3.5)	1 [Reference]
1-5	2 447 383 (43.5)	1 526 760	63.2 (63.2-63.3)	1.27 (1.26-1.29)	851 507	36.0 (35.9-36.0)	1.10 (1.09-1.11)	111 878	5.9 (5.9-6.0)	1.35 (1.31-1.38)
6-10	1 249 089 (22.2)	880 476	71.5 (71.4-71.6)	1.42 (1.41-1.43)	533 647	44.2 (44.1-44.3)	1.19 (1.18-1.20)	126 805	9.4 (9.3-9.4)	1.7 (1.66-1.75)
11-20	1 017 444 (18.1)	767 201	76.7 (76.6-76.8)	1.54 (1.53-1.55)	502 969	51.5 (51.4-51.6)	1.30 (1.29-1.3)	110 211	13.2 (13.1-13.3)	2.02 (1.96-2.07)
≥21	626 384 (11.1)	502 285	82.6 (82.6-82.8)	1.76 (1.75-1.77)	356 471	61.4 (61.2-61.5)	1.45 (1.43-1.46)	138 739	19.6 (19.4-19.6)	2.45 (2.39-2.52)

Abbreviations: aHR, adjusted hazard ratio; BMI, body mass index (calculated as weight in kilograms divided by height in meters squared); CCI, Charlson Comorbidity Index; COPD, chronic obstructive pulmonary disease; PTSD, posttraumatic stress disorder; VA, Department of Veterans Affairs; VISN, VA Integrated Service Network.

^a^ Veterans 18 years or older with an inpatient, outpatient, or telehealth encounter, including a primary care visit, in the VA health care system in the 24 months preceding cohort entry on December 1, 2020.

^b^ Adjusted for sex, age, race, ethnicity, urban vs rural residence, VISN, SARS-CoV-2 infection, BMI, CCI, number of primary care visits in 2 years before December 1, 2020, number of mental health visits in prior 2 years, and number of specialty care visits in prior 2 years.

^c^ Cumulative incidence among entire cohort.

^d^ Includes veterans who completed primary vaccination and obtained a first booster dose on or after September 22, 2021.

^e^ Includes veterans 50 years or older as of March 2022 who completed first booster vaccination and obtained a second booster dose on or after March 29, 2022.

^f^ Reference group for second booster includes veterans aged 18 to 49 years at the start of the study period in December 2020 who were 50 years or older as of March 2022 when second booster vaccination received US Food and Drug Administration Emergency Use Authorization.

^g^ Other race includes veterans who self-identified their race as other or who self-identified as belonging to more than 1 race category.

^h^ Based on Rural-Urban Commuting Area codes.

^i^ SARS-CoV-2 infection diagnosed before or on the date of completion of COVID-19 primary, first booster, second booster vaccination, or last day of observation period.

### COVID-19 Vaccination Definitions

We aggregated all administered vaccine doses documented in the CDW, CMS Medicare, and VA Community Care records. Vaccine records with service dates before December 11, 2020, the earliest date of EUA for COVID-19 vaccination in the US, were excluded. To ensure that vaccine doses documented in more than 1 source were not counted more than once, after combining records from all sources, we treated 2 vaccine doses as duplicates if they were documented as having been received within 7 days of each other.

We included mRNA-1273 (52% of all vaccine doses), BNT162b2 (45%), and JNJ-78436735 (3%) vaccine types, which were approved in the US during the period of study and comprised most of all vaccine types. To allow for complete ascertainment of vaccination in VHA, we also included AZD1222 (Oxford-AstraZeneca) (0.01%) and NVX-CoV2373 (<0.01%, authorized after the end of this study period). Vaccine doses of unknown or other type (0.01%) were excluded from the analysis.

Beginning December 1, 2020, we determined the time to completion of the following vaccination end points, with follow-up extending through June 30, 2022. Primary vaccination was indicated by receipt of 2 doses of mRNA (BNT162b2 or mRNA-1273), a single dose of JNJ-78436735, 2 doses of NVX-CoV2373, or 2 doses of AZD1222 COVID-19 vaccine. First booster vaccination was indicated by any primary vaccination regimen above, followed by an additional dose of mRNA, JNJ-78436735, NVX-CoV2373, or AZD1222 vaccine. Second booster vaccination was indicated by any first booster vaccination above, followed by an additional dose of mRNA, JNJ-78436735, NVX-CoV2373, or AZD1222 vaccine.

Heterologous vaccination (use of different vaccine products) was allowed for determination of primary and booster vaccination. Prior SARS-CoV-2 infection was based on the earlier of either first laboratory-confirmed SARS-CoV-2 test in CSDR or first COVID-19 diagnosis date in CMS Medicare data and ascertained as of the date of primary or booster vaccination completion.

### Statistical Analysis

Descriptive statistics were generated for patients receiving primary and booster vaccination. Kaplan-Meier curves were used to compare cumulative incidence of vaccination by selected characteristics. Sensitivity analysis was conducted from December 1, 2020, through December 31, ,2021 comparing VA data sources alone and combined VA and CMS Medicare data.

Cox proportional hazards regression was used to identify factors independently associated with time to primary and booster vaccination after adjusting for sex, age, race, ethnicity, urban vs rural residence, VISN, SARS-CoV-2 infection (modeled as a time-varying covariate), BMI, CCI, and number of primary care, mental health, or specialty care visits in the prior 2 years. We did not adjust for CCI when evaluating individual conditions to avoid overadjustment because the CCI quantifies numerous underlying conditions. Time-to-event analyses were censored on June 30, 2022, or at the time of death if earlier.

For primary vaccination, time-to-event Cox proportional hazards regression analyses began on December 11, 2020, and were conducted among all eligible cohort members. For first booster vaccination, time-to-event analyses began on September 22, 2021, when the FDA authorized a booster dose of the BNT162b2 COVID-19 vaccine, and were limited to cohort members who had completed primary vaccination.^3^ For second booster vaccination, time-to-event analyses began on March 29, 2022, when the FDA authorized a second booster vaccination, and were limited to cohort members 50 years or older as of March 2022 who had received a first booster. Cox proportional hazards regression analyses were limited to persons who were alive and had not yet experienced the vaccination outcome of interest as of the first day of follow-up. The proportional hazards assumption was tested using log-log plot and Schoenfeld residuals. Cumulative incidence and HRs were compared using 95% CIs. Analyses were conducted in Stata software, version 17 (StataCorp LLC).

## Results

### Cohort Characteristics

We identified 8 822 259 persons 18 years or older who were enrolled in VHA and alive as of December 1, 2020. We excluded 2 455 835 veterans (27.8%) without any inpatient, outpatient, or telehealth encounters in the preceding 24 months as well as an additional 734 011 veterans (8.3%) without primary care visits during the same period. Among the 5 632 413 veterans included in the study, 5 094 392 (90.4%) were male and 538 021 (9.6%) were female; the median (IQR) age was 66 (51-74) years; 64 574 (1.1%) were American Indian or Alaska Native, 80 031 (1.4%) were Asian, 1 032 334 (18.3%) were Black, 58 643 (1.0%) were Native Hawaiian or other Pacific Islander, 4 202 173 (74.6%) were White, 5900 (0.1%) were of other race, and 188 758 (3.4%) were missing race; 448 714 (8.0%) were Hispanic or Latino, 4 946 545 (87.8%) were not Hispanic or Lationo, and 237 154 (4.2%) were missing ethnicity (Table). The median (IQR) CCI was 1 (0-2), and 300 359 veterans (5.3%) had received immunosuppressive medications or cancer treatments. A total of 2 445 354 veterans (43.4%) had obesity, 1 520 589 (30.2%) had diabetes, 714 205 (12.7%) had chronic obstructive pulmonary disease (COPD), and 564 161 (10.0%) had chronic kidney disease.

### Cumulative Incidence of Vaccination

**Figure 1.**
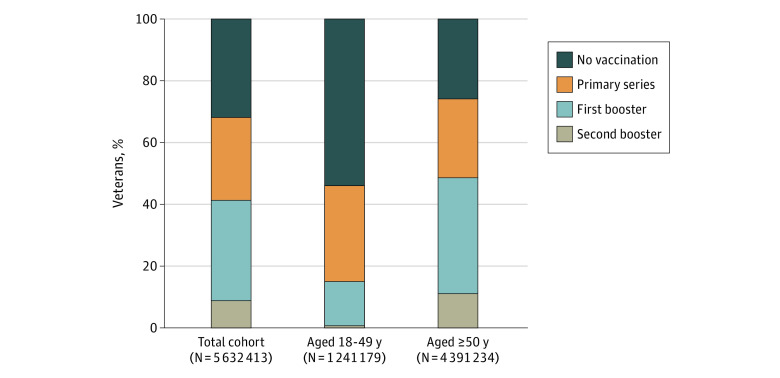
Percentage of US Veterans With No Receipt and Receipt of Primary and Booster Vaccination for COVID-19 as of June 30, 2022, Overall and by Age Group Age was determined in March 2022, when the US Food and Drug Administration authorized a second booster vaccination for persons 50 years or older.

**Figure 2.**
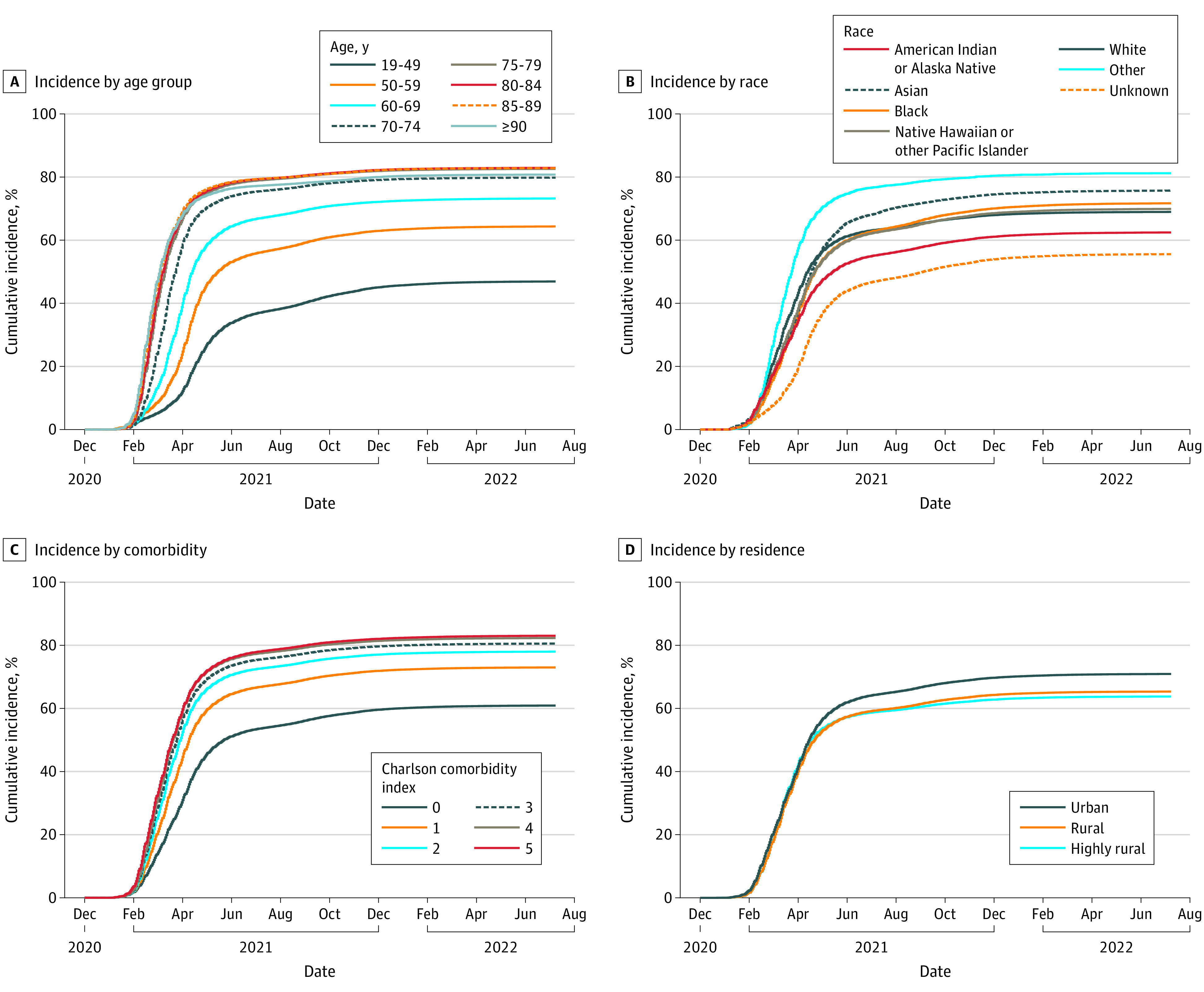
Cumulative Incidence of COVID-19 Primary Vaccination Among US Veterans, December 1, 2020, to June 30, 2022

**Figure 3.**
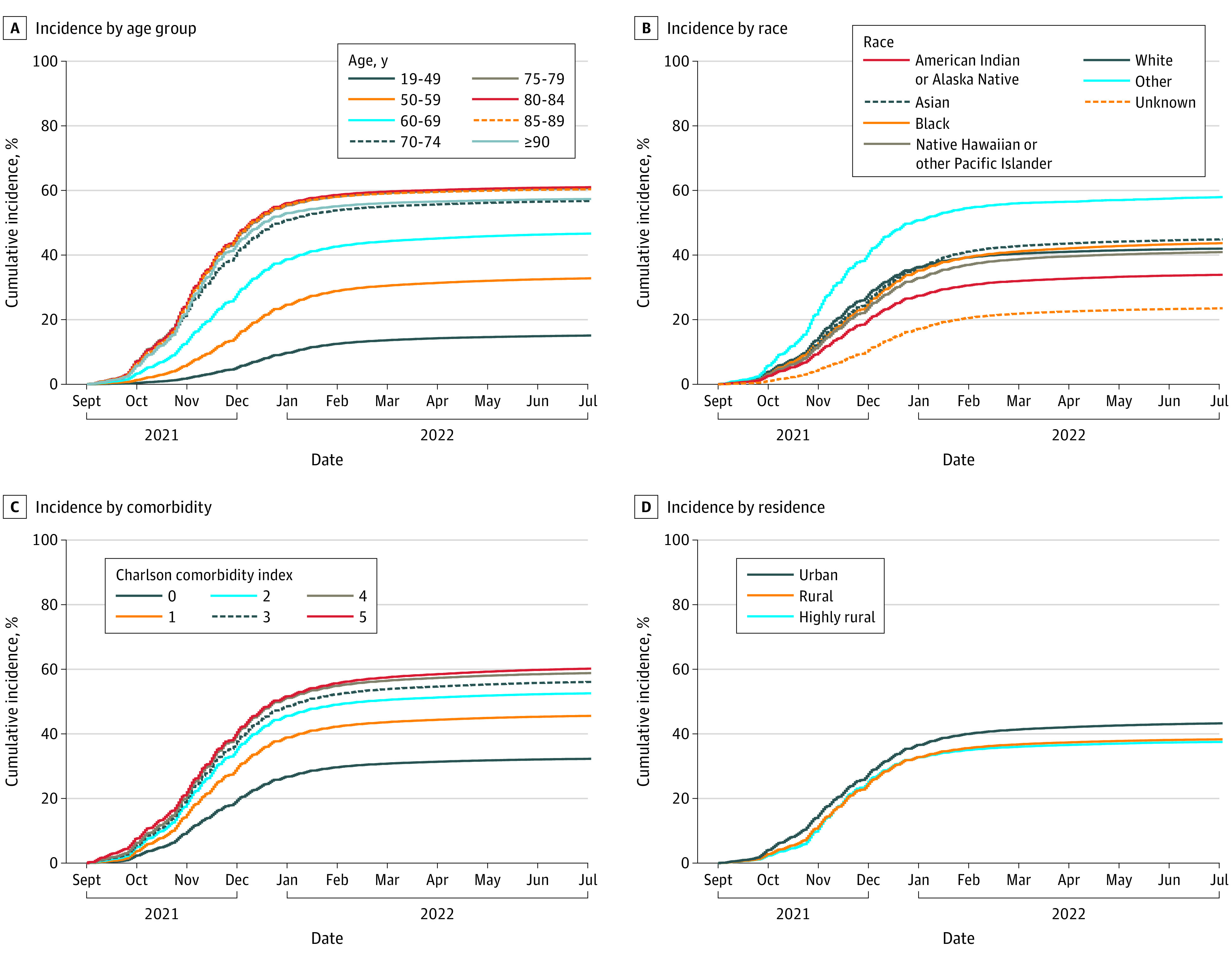
Cumulative Incidence of COVID-19 First Booster Vaccination Among US Veterans, September 1, 2021, to June 30, 2022

**Figure 4.**
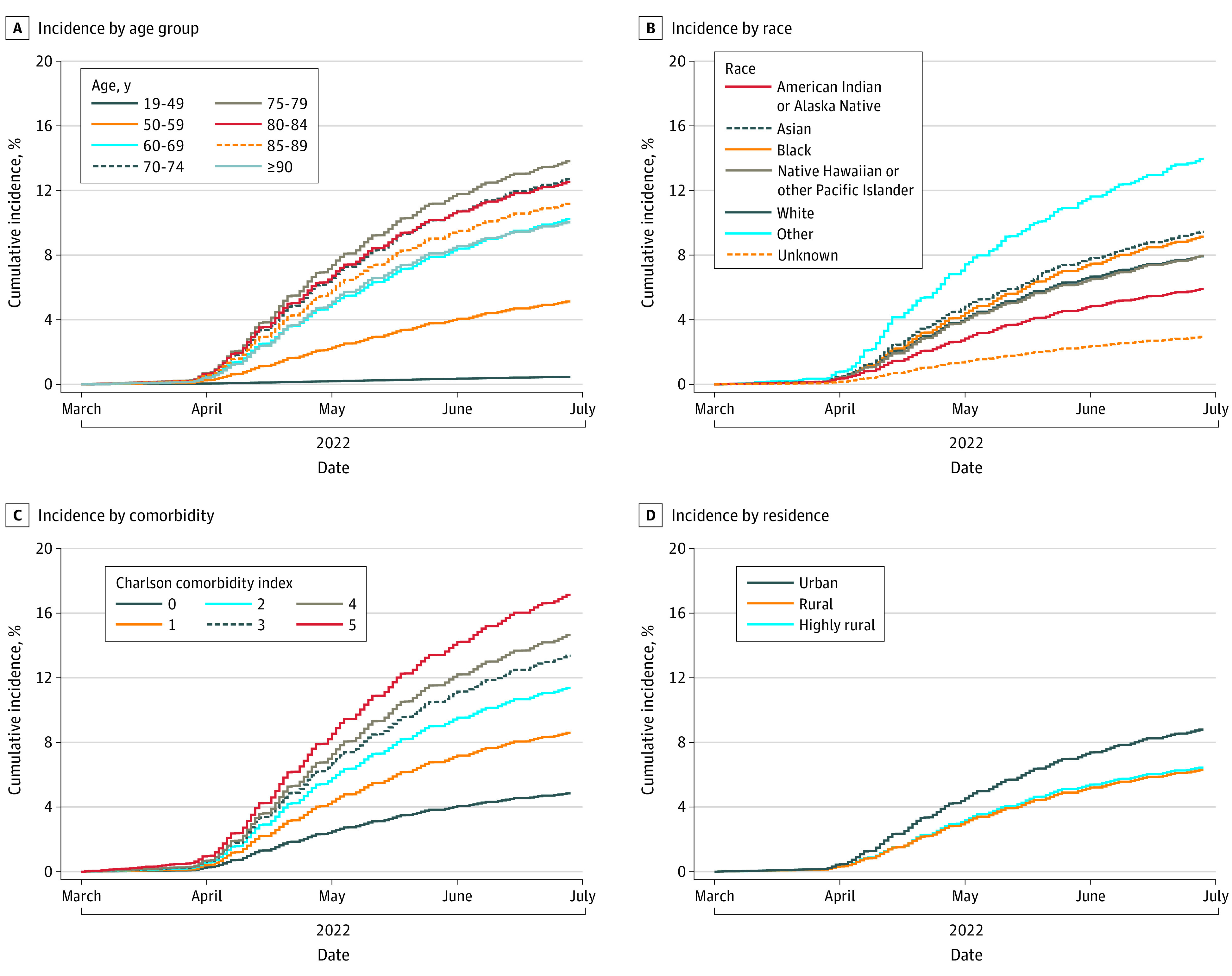
Cumulative Incidence of COVID-19 Second Booster Vaccination Among US Veterans, March 1, 2022, to June 30, 2022

By June 30, 2022, a total of 32.1% of veterans were unvaccinated, 26.7% had completed primary vaccination only (no booster), and 41.2% had completed booster vaccination (Figure 1); among veterans aged 18 to 49 years, 54.0% were unvaccinated and 31.1% had completed primary vaccination only. Cumulative vaccination incidences were 69.0% for primary vaccination, 42.9% for first booster, and 9.3% for second booster (Table). Cumulative incidence for primary vaccination increased with age and was 46.9% (95% CI, 46.8%-47.0%) among veterans aged 18 to 49 years, 73.2% (95% CI, 73.1%-73.3%) among veterans aged 60 to 69 years, and 82.9% (95% CI, 82.8%-83.0%) among veterans aged 80-84 years (Table and Figure 2); similarly, for a first booster it increased from 15.7% (95% CI, 15.7%-15.8%) among veterans aged 18 to 49 years to 47.7% (95% CI, 47.6%-47.8%) among veterans aged 60 to 69 years to 62.4% (95% CI, 62.3%-62.6%) among veterans aged 80-84 years and for a second booster from 11.4% (95% CI, 11.3%-11.4%) among veterans aged 60 to 69 years to 14.9% (95% CI, 14.7%-15.0%) for veterans aged 80 to 84 years (Table, Figure 3 and Figure 4).

More veterans of Black race completed primary vaccination (71.7%; 95% CI, 71.6%-71.8%) compared with White veterans (68.9%; 95% CI, 68.9%-69.0%). Completion of primary vaccination was also higher among Asian compared with White veterans (75.7%; 95% CI, 75.4%-76.0%). Incidence was lowest among American Indian and Alaska Native groups (primary vaccination: 62.4%; 95% CI, 62.1%-62.8%).

Cumulative incidence of primary and booster vaccination was higher among veterans who had not experienced prior SARS-CoV-2 infection. Incidence increased with higher CCI; cumulative incidences for primary vaccination were 60.9% for veterans with a CCI of 0, 73.0% for those with a CCI of 1, 78.0% for a CCI of 2, and 83.0% for a CCI of 5 or higher; for first booster vaccination, 33.3%, 46.7%, 53.9%, and 61.9%, respectively; and for second booster vaccination, 5.9%, 10.1%, 13.1%, and 18.9%, respectively. Cumulative incidence of primary, first booster, and second booster vaccination was higher for veterans with conditions such as diabetes, chronic kidney disease, and COPD compared with those without the respective conditions but lower for mental health conditions, including major depressive disorder, posttraumatic stress disorder, and bipolar disorder or schizophrenia (Table). Cumulative incidence of vaccination was also higher for veterans with higher numbers of primary and specialty care visits (eFigure in Supplement 1). The absolute increase in incidence with the addition of CMS Medicare data was small (eTable 2 in Supplement 1).

### Factors Associated With Vaccination

Adjusting for demographic, clinical, and geographic characteristics (Table), vaccination was more likely among older age groups (for age 80-84 years vs 18-49 years, primary adjusted hazard ratio [aHR], 3.94; 95% CI, 3.92-3.96; first booster aHR, 3.68; 95% CI, 3.65-3.70; second booster aHR, 2.75; 95% CI, 2.62-2.88) and women (vs men, primary aHR, 1.07; 95% CI, 1.06-1.07; first booster aHR, 1.09; 95% CI, 1.08-1.09; second booster aHR, 1.10; 95% CI, 1.09-1.12). Black veterans were more likely than White veterans to receive vaccination (primary aHR, 1.10; 95% CI, 1.11-1.12; first booster aHR, 1.15; 95% CI, 1.14-1.15; second booster aHR, 1.24; 95% CI, 1.23-1.25), as were Asian persons (primary aHR, 1.48; 95% CI, 1.46-1.49; first booster aHR, 1.36; 95% CI, 1.34-1.37; second booster aHR, 1.47; 95% CI, 1.44-1.51) and Hispanic compared with non-Hispanic veterans (primary aHR, 1.11; 95% CI, 1.11-1.12; first booster aHR, 1.07; 95% CI, 1.06-1.08; second booster aHR, 1.11; 95% CI, 1.09-1.12). American Indian and Alaska Native groups were less likely to receive vaccination compared with White veterans (primary aHR, 0.95; 95% CI, 0.94-0.96; first booster aHR, 0.96; 95% CI, 0.95-0.98).

Veterans living in highly rural areas were less likely (63.8%; 95% CI, 63.4%-64.1%) to complete vaccination than urban veterans (primary aHR, 0.70; 95% CI, 0.70-0.71 highly rural; aHR, 0.81; 95% CI, 0.81-0.81 rural). Persons experiencing prior SARS-CoV-2 infection were less likely to receive any vaccination (primary aHR, 0.37; 95% CI, 0.37-0.38; first booster aHR, 0.53; 95% CI, 0.53-0.53; second booster aHR, 0.73; 95% CI, 0.73-0.74).

Veterans with higher CCIs (≥5 vs 0) were more likely to have primary vaccination (aHR, 1.09; 95% CI, 1.08-1.09) but less likely to have a first booster (aHR, 0.85; 95% CI, 0.85-0.86). Likelihood of primary vaccination also varied by individual underlying condition (diabetes: aHR, 1.06; 95% CI, 1.06-1.06; COPD: aHR, 0.98; 95% CI, 0.98-0.98). Higher numbers of baseline primary and specialty care encounters were also associated with primary vaccination (≥12 vs 1-5 primary care visits: aHR, 1.10; 95% CI, 1.10-1.11; ≥21 vs no specialty care visits: aHR, 1.76; 95% CI, 1.75-1.77).

## Discussion

In this nationwide study of 5.6 million US veterans receiving VHA care as of December 2020, the cumulative vaccination incidences through June 2022 were 69.0% for primary vaccination, 42.9% for first booster, and 9.3% for second booster. Incidence was lowest among the youngest veterans (aged 18-49 years), with 46.9% receiving primary vaccination and 15.7% receiving first booster vaccination compared with older groups (82.9% receiving primary vaccination and 62.4% receiving first booster vaccination among veterans aged 80-84 years). Incidence was also higher among veterans living in urban compared with rural areas. There was geographic variability in incidence of vaccination across VA VISNs. Accounting for demographic, clinical, and geographic differences, likelihood of primary and booster vaccination was also much higher among older veterans as well as women; Asian, Black, and Hispanic groups; and residents of urban areas.

Although COVID-19 vaccines have been demonstrated to be safe and effective, rates of vaccination across the US remain suboptimal, and booster vaccination lags well behind primary vaccination despite being broadly recommended.^2,6,21,22^ Although national-level surveillance provides data on demographic and geographic trends, a more complete picture that includes important clinical factors is important to informing strategies to improve vaccination access and uptake. In this study, we present the most comprehensive, longitudinal assessment of COVID-19 vaccination across VHA to date and incorporate detailed background on demographic, geographic, and clinical factors, including health care use and underlying conditions.

As observed in the general US population, compared with older veterans, we found that uptake of vaccination among younger veterans has remained lower.^6^ More than half (54.0%) of veterans in care aged 18 to 49 years were unvaccinated, whereas 31.1% had completed primary vaccination only. Even among veterans aged 50 to 69 years, incidence was still significantly lower than in older groups. Differences in risk perception of SARS-CoV-2 infection and related outcomes, vaccine safety and efficacy, and trust in authorities may contribute to a lower incidence and likelihood of vaccination among younger groups.^23^

Notable differences were seen in vaccination by racial and ethnic groups, with higher incidence among Asian, Black, and Hispanic veterans compared with White and non-Hispanic groups. Following observation of early disparities in vaccination coverage among US racial and ethnic minorities, efforts to provide equitable vaccine access were undertaken to reduce this gap.^7^ In VHA, during the early months after initial COVID-19 vaccine EUA, vaccination coverage was higher among Black and Hispanic veterans compared with non-Hispanic White veterans.^24^ Equal access to comprehensive VHA health care and targeted outreach mitigates some racial and ethnic disparities.^25^ Consistent with a previous study,^7^ we found that incidence of vaccination has remained lower for American Indian and Alaska Native groups. This finding is particularly concerning because American Indian and Alaska Native persons have been disproportionately affected by COVID-19, including higher rates of illness, hospitalization, and death.^26,27^ Continued support and targeted outreach for COVID-19 vaccination within VHA remain important to reduce these disparities.

Differences in COVID-19 vaccination by rurality were also observed, with veterans in urban areas more likely to complete primary and booster vaccination. Although the cumulative incidence of primary and first booster vaccination in this study was 5% higher among urban residents, this difference is smaller than in nonveteran populations, where the gap has increased significantly over time.^28^ Possible reasons for observed differences include dedicated programmatic efforts to provide care to rural veterans as well as the role of VHA and VHA practitioners as trusted sources of vaccine information.^29,30^

After adjusting for demographic, clinical, and geographic characteristics, persons with a greater burden of underlying medical conditions, as measured by the CCI, had a slightly higher likelihood of primary vaccination but slightly lower likelihood of booster vaccination. In contrast, an earlier VA study^24^ found that the likelihood of vaccination during the first 3 months following COVID-19 vaccine EUA increased significantly among persons with higher CCIs. Although early vaccination efforts prioritized persons at highest risk for severe COVID-19 outcomes, these results indicate that, over time, higher-risk patients may have less impetus for receiving COVID-19 vaccination, despite their increased risk of severe COVID-19 outcomes.^31^

### Limitations

This study has several limitations. First, although we restricted the study population to veterans engaged in care and integrated multiple data sources to strengthen ascertainment of COVID-19 vaccination, we likely did not fully capture all vaccinations received outside VHA. In addition, CMS Medicare vaccination data only extended to December 31, 2021; however, we demonstrated in sensitivity analysis that the overall absolute increase in vaccination uptake with the addition of these data was relatively small. Underascertainment of vaccination is likely to be more pronounced for booster vaccination because vaccines have become more broadly accessible in the community over time. Although the absolute vaccination rates that we report underestimate slightly the true vaccination rates of VHA enrollees, the associations and trends of different vaccination factors are likely to be consistent. Second, we did not specify primary vaccination separately for immunocompromised persons, for whom additional vaccine doses are recommend^32^; however, overall misclassification of primary vs booster vaccination in this study is expected to be low given the relatively low prevalence in the cohort. Third, our definitions of primary and booster vaccination did not require prespecified intervals between doses,^2^ which may have resulted in a small degree of overascertainment of vaccination completion. In a prior VA study,^24^ we demonstrated that a second mRNA vaccine dose was administered within 4 days of the recommended date in 95% of cases for both BNT162b2and mRNA-1273, demonstrating excellent dosing adherence. On the other hand, we considered as duplicates any 2 vaccine doses administered within 7 days of each other, whereas other estimates did not apply this criterion.^6^ Comparisons of vaccination coverage reported in VA vs non-VA studies must therefore be interpreted with caution.

## Conclusions

In this retrospective cohort study of US veterans receiving VHA care, uptake of COVID-19 primary and booster vaccination remained underused, similar to trends observed in the general US population. Several important demographic and clinical factors were associated with vaccination. Younger, rural, American Indian and Alaska Native groups, and persons with a high burden of underlying conditions may benefit from targeted outreach to improve COVID-19 vaccination rates.

## Notes

Supplement 1. **eTable 1.** Key Relevant COVID-19 Vaccine U.S. Food and Drug Administration Emergency Use Authorization Dates, December 2020-March 2022 **eTable 2.** Cumulative Incidence of COVID-19 Primary and First Booster Vaccination Among VA Enrollees^a^ Based on VA Data Sources^b^ vs VA Data Combined With CMS-Medicare Data Sources, December 2020-December 2021 **eFigure.** Cumulative Incidence of COVID-19 Primary Vaccination (A-B), First Booster (C-D), and Second Booster Vaccination (E-F) Among U.S. Veterans by Number of Primary and Specialty Care Visits in the Prior 2 Years, December 1, 2020-June 30, 2022

Supplement 2.
Data Sharing Statement

## References

[1] Fleming-Dutra KE, Wallace M, Moulia DL, . Interim Recommendations of the Advisory Committee on Immunization Practices for Use of Moderna and Pfizer-BioNTech COVID-19 Vaccines in Children Aged 6 Months-5 Years - United States, June 2022. MMWR Morb Mortal Wkly Rep. 2022;71(26):859-868. doi:10.15585/mmwr.mm7126e2 35771731

[2] Centers for Disease Control and Prevention. COVID-19 Vaccine: Interim COVID-19 Immunization Schedule for Persons 6 Months of Age and Older. Accessed November 29, 2022. https://www.cdc.gov/vaccines/covid-19/downloads/COVID-19-immunization-schedule-ages-6months-older.pdf

[3] US Food and Drug Administration. COVID-19 Vaccines. Accessed July 16, 2022. https://www.fda.gov/emergency-preparedness-and-response/coronavirus-disease-2019-covid-19/covid-19-vaccines#authorized-vaccines

[4] Centers for Disease Control and Prevention. ACIP Update to the Evidence to Recommendations for a 2nd COVID-19 Booster Dose in Adults Ages 50 Years and Older and Immunocompromised Individuals. Accessed August 3, 2022. https://www.cdc.gov/vaccines/acip/recs/grade/covid-19-second-booster-dose-etr.html

[5] Centers for Disease Control and Prevention. Use of COVID-19 Vaccines in the United States: US Department of Health & Human Services. Accessed December 2, 2022. https://www.cdc.gov/vaccines/covid-19/clinical-considerations/covid-19-vaccines-us.html

[6] Centers for Disease Control and Prevention. COVID-19 Vaccinations in the United States. Accessed July 16, 2022. https://covid.cdc.gov/covid-data-tracker/#vaccinations_vacc-people-additional-dose-totalpop

[7] Kriss JL, Hung MC, Srivastav A, . COVID-19 Vaccination coverage, by race and ethnicity—national immunization survey adult COVID module, United States, December 2020-November 2021. MMWR Morb Mortal Wkly Rep. 2022;71(23):757-763. doi:10.15585/mmwr.mm7123a2 35679179 PMC9181054

[8] US Department of Veterans Affairs. Veterans Health Administration. Accessed March 1, 2022. https://www.va.gov/health/

[9] US Department of Veterans Affairs. Veterans Affairs Corporate Data Warehouse. Accessed March 22, 2021. https://www.hsrd.research.va.gov/for_researchers/vinci/cdw.cfm

[10] US Department of Veterans Affairs. COVID-19: Shared Data Resource. Accessed March 22, 2021. https://vhacdwdwhweb100.vha.med.va.gov/phenotype/index.php/COVID-19:Shared_Data_Resource

[11] US Department of Veterans Affairs. Community Care. Accessed July 16, 2022. https://www.va.gov/communitycare/

[12] Veterans Health Administration. Veterans Integrated Services Networks (VISNs). Accessed May 10, 2022. https://www.va.gov/HEALTH/visns.asp?msclkid=5dbaec00d09811ec9cf829deb0e7f5ef

[13] US Department of Agriculture. Rural-Urban Commuting Area Codes. Accessed May 10, 2022. https://www.ers.usda.gov/data-products/rural-urban-commuting-area-codes/

[14] US Department of Veterans Affairs. Rural Veterans. Accessed August 8, 2022. https://www.ruralhealth.va.gov/aboutus/ruralvets.asp#def

[15] Ioannou GN, Locke E, Green P, . Risk factors for hospitalization, mechanical ventilation, or death among 10 131 US veterans with SARS-CoV-2 infection. JAMA Netw Open. 2020;3(9):e2022310. doi:10.1001/jamanetworkopen.2020.22310 32965502 PMC7512055

[16] Kompaniyets L, Pennington AF, Goodman AB, . Underlying medical conditions and severe illness among 540,667 adults hospitalized with COVID-19, March 2020-March 2021. Prev Chronic Dis. 2021;18:E66. doi:10.5888/pcd18.210123 34197283 PMC8269743

[17] Deyo RA, Cherkin DC, Ciol MA. Adapting a clinical comorbidity index for use with ICD-9-CM administrative databases. J Clin Epidemiol. 1992;45(6):613-619. doi:10.1016/0895-4356(92)90133-8 1607900

[18] Charlson ME, Pompei P, Ales KL, MacKenzie CR. A new method of classifying prognostic comorbidity in longitudinal studies: development and validation. J Chronic Dis. 1987;40(5):373-383. doi:10.1016/0021-9681(87)90171-8 3558716

[19] Bajema KL, Wang XQ, Hynes DM, . Early adoption of anti-SARS-CoV-2 pharmacotherapies among US veterans With mild to moderate COVID-19, January and February 2022. JAMA Netw Open. 2022;5(11):e2241434-e2241434. doi:10.1001/jamanetworkopen.2022.41434 36367727 PMC9652752

[20] Ioannou GN, Baraff A, Fox A, . Rates and factors associated with documentation of diagnostic codes for long COVID in the National Veterans Affairs Health Care System. JAMA Netw Open. 2022;5(7):e2224359. doi:10.1001/jamanetworkopen.2022.24359 35904783 PMC9338411

[21] Centers for Disease Control and Prevention. Ensuring COVID-19 Vaccine Safety in the US. Accessed August 6, 2022. https://www.cdc.gov/coronavirus/2019-ncov/vaccines/safety.html

[22] Moreira ED Jr, Kitchin N, Xu X, ; C4591031 Clinical Trial Group. Safety and efficacy of a third dose of BNT162b2 Covid-19 vaccine. N Engl J Med. 2022;386(20):1910-1921. doi:10.1056/NEJMoa2200674 35320659 PMC9006787

[23] Joshi A, Kaur M, Kaur R, Grover A, Nash D, El-Mohandes A. Predictors of COVID-19 vaccine acceptance, intention, and hesitancy: a scoping review. Front Public Health. 2021;9:698111. doi:10.3389/fpubh.2021.698111 34485229 PMC8414566

[24] Ioannou GN, Green P, Locke ER, Berry K. Factors associated with early receipt of COVID-19 vaccination and adherence to second dose in the Veterans Affairs healthcare system. PLoS One. 2021;16(12):e0259696. doi:10.1371/journal.pone.0259696 34851970 PMC8635372

[25] Peterson K, Anderson J, Boundy E, Ferguson L, McCleery E, Waldrip K. Mortality disparities in racial/ethnic minority groups in the Veterans Health Administration: an evidence review and map. Am J Public Health. 2018;108(3):e1-e11. doi:10.2105/AJPH.2017.304246 29412713 PMC5803811

[26] Ward LA, Black KP, Britton CL, Tompkins ML, Provost EM. COVID-19 cases, hospitalizations, and deaths among American Indian or Alaska Native persons—Alaska, 2020-2021. MMWR Morb Mortal Wkly Rep. 2022;71(22):730-733. doi:10.15585/mmwr.mm7122a2 35653289 PMC9169521

[27] Hatcher SM, Agnew-Brune C, Anderson M, . COVID-19 among American Indian and Alaska Native persons—23 States, January 31-July 3, 2020. MMWR Morb Mortal Wkly Rep. 2020;69(34):1166-1169. doi:10.15585/mmwr.mm6934e1 32853193 PMC7451969

[28] Saelee R, Zell E, Murthy BP, . Disparities in COVID-19 vaccination coverage between urban and rural counties—United States, December 14, 2020-January 31, 2022. MMWR Morb Mortal Wkly Rep. 2022;71(9):335-340. doi:10.15585/mmwr.mm7109a2 35239636 PMC8893338

[29] US Department of Veterans Affairs. VHA Office of Rural Health. Accessed August 7, 2022. https://www.ruralhealth.va.gov/

[30] Jasuja GK, Meterko M, Bradshaw LD, . Attitudes and intentions of US veterans regarding COVID-19 vaccination. JAMA Netw Open. 2021;4(11):e2132548. doi:10.1001/jamanetworkopen.2021.32548 34730819 PMC8567110

[31] Yek C, Warner S, Wiltz JL, . Risk factors for severe COVID-19 outcomes among persons aged ≥18 years who completed a primary COVID-19 vaccination series—465 health care facilities, United States, December 2020-October 2021. MMWR Morb Mortal Wkly Rep. 2022;71(1):19-25. doi:10.15585/mmwr.mm7101a4 34990440 PMC8735560

[32] Centers for Disease Control and Prevention. COVID-19 vaccination guidance for people who are moderately or severely immunocompromised. Accessed August 7, 2022. https://www.cdc.gov/vaccines/covid-19/clinical-considerations/interim-considerations-us.html#immunocompromised

