# Supplementary material for: Rates of and Factors Associated With Primary and Booster COVID-19 Vaccine Receipt by US Veterans, December 2020 to June 2022
Source: JAMA Netw Open. 2023 Feb 2;6(2):e2254387. doi: 10.1001/jamanetworkopen.2022.54387 (PMC9896301; doi:10.1001/jamanetworkopen.2022.54387)
Supplement: Supplement 1. — eTable 1. Key Relevant COVID-19 Vaccine U.S. Food and Drug Administration Emergency Use Authorization Dates, December 2020-March 2022 eTable 2. Cumulative Incidence of COVID-19 Primary and First Booster Vaccination Among VA Enrolleesa Based on VA Data Sourcesb vs VA Data Combined With CMS-Medicare Data Sources, December 2020-December 2021 eFigure. Cumulative Incidence of COVID-19 Primary Vaccination (A-B), First Booster (C-D), and Second Booster Vaccination (E-F) Among U.S. Veterans by Number of Primary and Specialty Care Visits in the Prior 2 Years, December 1, 2020-June 30, 2022 [file jamanetwopen-e2254387-s001.pdf]

Supplementary Online Content

Bajema KL, Rowneki M, Berry K, et al. Rates of and factors associated with primary and booster COVID-19 vaccine receipt by US veterans, December 2020 to June 2022. *JAMA Netw Open*. 2023;6(2):e2254387. doi:10.1001/jamanetworkopen.2022.54387

eTable 1. Key Relevant COVID-19 Vaccine U.S. Food and Drug Administration Emergency Use Authorization Dates, December 2020-March 2022

eTable 2. Cumulative Incidence of COVID-19 Primary and First Booster Vaccination Among VA Enrollees[a] Based on VA Data Sources[b] vs VA Data Combined With CMS-Medicare Data Sources, December 2020-December 2021

eFigure. Cumulative Incidence of COVID-19 Primary Vaccination (A-B), First Booster (C-D), and Second Booster Vaccination (E-F) Among U.S. Veterans by Number of Primary and Specialty Care Visits in the Prior 2 Years, December 1, 2020-June 30, 2022

This supplementary material has been provided by the authors to give readers additional information about their work.

© 2023 Bajema KL et al. *JAMA Network Open.*

eTable 1. Key Relevant COVID-19 Vaccine U.S. Food and Drug Administration Emergency Use Authorization Dates, December 2020-March 2022

Date of EUA Issuance	Authorized Use
December 11, 2020	Pfizer-BioNTech authorized for persons aged ≥16 years
December 18, 2020	Moderna authorized for persons aged ≥18 years
February 27, 2021	Janssen (Johnson & Johnson) authorized for persons aged ≥18 years
August 12, 2021	Third dose of Pfizer-BioNTech and Moderna authorized for certain immunocompromised persons aged ≥18 years
September 22, 2021	Pfizer-BioNTech booster authorized for certain individuals:<br>- Aged ≥65 years<br>- Aged 18-64 years and at high risk for severe COVID-19<br>- Age 18-64 years and at high risk of serious complications of COVID-19 due to frequent institutional or occupational exposure to SARS-CoV-2
October 20, 2021	Moderna and Janssen boosters authorized for certain individuals (as noted above)
November 19, 2021	Pfizer-BioNTech and Moderna boosters expanded to include all persons aged ≥18 years
March 29, 2022	Pfizer-BioNTech and Moderna second boosters authorized for persons aged ≥50 years and persons with certain kinds of immunocompromise (Pfizer-BioNTech aged ≥12 years, Moderna aged ≥18 years)

Abbreviations: EUA, Emergency Use Authorization

© 2023 Bajema KL et al. *JAMA Network Open.*

eTable 2. Cumulative Incidence of COVID-19 Primary and First Booster Vaccination Among VA Enrollees[a] Based on VA Data Sources[b] vs VA Data Combined With CMS-Medicare Data Sources, December 2020-December 2021

		Primary vaccination		First Booster	
		VA Data Sources	Combined VA and CMS-Medicare Data Sources	VA Data Sources	Combined VA and CMS-Medicare Data Sources
	Veterans in Care at VA N (%)	Cumulative Incidence, % (95% CI)[c]	Cumulative Incidence, % (95% CI)[c]	Cumulative Incidence, % (95% CI)[c]	Cumulative Incidence, % (95% CI)[c]
**Overall**	5,632,413 (100)	65.4 (65.4-65.4)	68.4 (68.3-68.4)	30.2 (30.2-30.3)	36.7 (36.7-36.8)
**Sex**					
Female	538,021 (9.6)	61.6 (61.5-61.7)	62.6 (62.4-62.7)	24.5 (24.4-24.7)	26.4 (26.2-26.5)
Male	5,094,392 (90.5)	65.8 (65.8-65.8)	69.0 (68.9-69.0)	30.9 (30.8-30.9)	37.8 (37.8-37.9)
**Age (Years)**					
18-49	1,327,019 (23.6)	45.7 (45.6-45.8)	45.8 (45.7-45.9)	10.3 (10.3-10.4)	10.4 (10.4-10.5)
50-59	807,261 (14.3)	63.1 (63.0-63.2)	63.5 (63.4-63.6)	25.1 (25.0-25.2)	25.7 (25.6-25.8)
60-69	1,109,186 (19.7)	70.4 (70.3-70.4)	72.6 (72.5-72.6)	35.7 (35.6-35.8)	39.9 (39.8-40.0)
70-74	1,065,086 (18.9)	75.0 (74.9-75.1)	79.4 (79.3-79.5)	41.6 (41.5-41.7)	52.3 (52.2-52.4)
75-79	598,366 (10.6)	77.2 (77.1-77.3)	82.2 (82.1-82.3)	43.8 (43.6-43.9)	57.0 (56.8-57.1)
80-84	307,600 (5.5)	75.6 (75.4-75.7)	82.5 (82.4-82.7)	41.5 (41.3-41.6)	57.7 (57.5-57.9)
85-89	249,994 (4.4)	73.2 (73.0-73.4)	82.5 (82.3-82.6)	38.1 (37.9-38.3)	57.3 (57.1-57.5)
≥90	167,901 (3.0)	67.2 (67.0-67.5)	80.4 (80.2-80.6)	33.3 (33.0-33.5)	54.6 (54.3-54.9)
**Race**					

© 2023 Bajema KL et al. *JAMA Network Open.*

American Indian or Alaska Native	64,574 (1.1)	59.2 (58.8-59.6)	61.7 (61.3-62.0)	24.7 (24.3-25.0)	28.7 (28.3-29.0)
Asian	80,031 (1.4)	73.6 (73.3-73.9)	74.9 (74.6-75.3)	33.5 (33.2-33.8)	37.5 (37.1-37.8)
Black	1,032,334 (18.3)	69.0 (68.9-69.1)	70.7 (70.6-70.8)	33.1 (33.0-33.2)	36.4 (36.3-36.5)
Native Hawaiian or Other Pacific Islander	58,643 (1.0)	67.0 (66.6-67.4)	69.1 (68.7-69.4)	29.6 (29.3-30.0)	34.3 (33.9-34.7)
White	4,202,173 (74.6)	64.9 (64.8-64.9)	68.4 (68.3-68.4)	30.1 (30.1-30.2)	37.8 (37.7-37.8)
Other	5,900 (0.1)	76.9 (75.8-78.0)	80.7 (79.7-81.7)	42.6 (41.3-43.9)	52.1 (50.9-53.5)
Missing	188,758 (3.4)	54.4 (54.2-54.6)	54.6 (54.4-54.8)	17.7 (17.5-17.9)	18.1 (17.9-18.2)
**Ethnicity**					
Hispanic or Latino	448,714 (8.0)	68.6 (68.5-68.7)	70.0 (69.9-70.1)	31.2 (31.1-31.4)	34.9 (34.7-35.0)
Not Hispanic or Latino	4,946,545 (87.8)	65.3 (65.3-65.4)	68.4 (68.4-68.4)	30.4 (30.3-30.4)	37.2 (37.0-37.1)
Missing	237,154 (4.2)	60.9 (60.7-61.1)	64.6 (64.4-64.8)	25.9 (25.7-26.0)	32.9 (32.7-33.1)
**Urban/Rural**[g]					
Urban	3,771,263 (67.0)	67.5 (67.5-67.6)	70.2 (70.1-70.2)	31.8 (31.8-31.9)	38.0 (38.0-38.1)
Rural	1,769,184 (31.4)	61.2 (61.1-61.3)	64.7 (64.7-64.8)	27.0 (27.0-27.2)	34.2 (34.0-34.1)
Highly Rural	79,790 (1.4)	59.3 (59.0-59.7)	63.2 (62.9-63.6)	27.0 (26.7-27.3)	33.9 (33.6-34.2)
Missing	12,176 (0.2)	62.5 (61.6-63.4)	63.0 (62.2-63.9)	28.2 (27.4-29.0)	28.8 (28.0-29.6)
**VA Region**[d]					
Midwest	1,101,919 (19.6)	65.5 (65.4-65.6)	69.3 (69.2-69.4)	33.9 (33.8-34.0)	41.5 (41.5-41.6)
Northeast	902,660 (16.0)	68.7 (68.7-68.8)	72.4 (72.3-72.5)	34.7 (34.6-34.8)	42.6 (42.5-42.7)
South	2,291,225 (40.7)	63.4 (63.3-63.5)	66.6 (66.1-66.2)	27.1 (27.1-27.2)	33.1 (33.1-33.2)
West	1,276,997 (22.7)	65.8 (65.7-65.9)	68.0 (67.9-68.1)	28.7 (28.6-28.8)	34.2 (34.1-34.3)

© 2023 Bajema KL et al. *JAMA Network Open.*

Missing	58,612 (1.1)	81.7 (81.4-82.0)	83.1 (82.8-83.4)	47.4 (47.0-47.8)	50.5 (50.1-50.9)
**Body mass index (kg/m²)**					
<18.5	47,330 (0.8)	58.8 (58.4-59.3)	63.1 (62.6-63.5)	24.6 (24.2-25.0)	29.6 (29.2-30.0)
18.5 to <25	1,015,185 (18.0)	62.7 (62.6-62.8)	66.4 (66.3-66.5)	28.6 (28.5-28.7)	35.7 (35.6-35.8)
Overweight	1,982,640 (35.2)	66.1 (66.1-66.2)	69.3 (69.2-69.4)	31.3 (31.2-31.3)	38.6 (38.6-38.7)
Obese I	1,469,359 (26.1)	66.8 (66.7-66.8)	69.3 (69.3-69.4)	31.3 (31.2-31.3)	37.3 (37.2-37.4)
Obese II	646,630 (11.5)	67.1 (66.9-67.2)	69.3 (69.1-69.4)	30.8 (30.7-30.9)	35.9 (35.8-36.0)
Obese III	329,365 (5.9)	67.0 (66.8-67.2)	69.1 (68.9-69.2)	29.9 (29.7-30.0)	34.1 (34.0-34.3)
Missing	141,904 (2.5)	50.4 (50.1-50.6)	54.8 (54.5-55.0)	16.5 (16.3-16.7)	22.7 (22.5-22.9)
**Charlson Comorbidity Index (CCI)**					
0	2,789,879 (49.5)	57.7 (57.6-57.7)	60.1 (60.1-60.2)	22.7 (22.6-22.7)	27.9 (27.8-27.9)
1	1,143,086 (20.3)	69.0 (68.9-69.0)	72.3 (72.3-72.4)	32.7 (32.6-32.8)	40.2 (40.1-40.3)
2	754,978 (13.4)	73.8 (73.7-73.9)	77.4 (77.3-77.5)	38.6 (38.5-38.7)	47.1 (47.0-47.2)
3	376,787 (6.7)	76.3 (76.2-76.5)	80.0 (79.9-80.1)	41.9 (41.7-42.1)	50.2 (50.0-50.3)
4	238,746 (4.2)	78.1 (77.9-78.3)	81.8 (81.6-81.9)	44.6 (44.4-44.8)	52.8 (52.6-53.0)
≥5	328,937 (5.8)	79.5 (79.3-79.6)	82.4 (82.3-82.6)	47.7 (47.5-47.9)	53.8 (53.6-53.9)
**Chronic Kidney Disease**					
No	5,068,252 (90.0)	64.2 (64.1-64.2)	67.0 (67.0-67.1)	29.0 (28.9-29.0)	35.2 (35.2-35.2)

© 2023 Bajema KL et al. *JAMA Network Open.*

Yes	564,161 (10.0)	76.6 (76.5-76.8)	80.6 (80.5-80.7)	42.5 (42.4-42.6)	51.4 (51.2-51.5)
**Chronic Obstructive Pulmonary Disease**					
No	4,918,208 (87.3)	64.2 (64.2-64.3)	67.1 (67.1-67.2)	29.0 (29.0-29.1)	35.4 (35.4-35.5)
Yes	714,205 (12.7)	73.6 (73.5-73.7)	77.0 (76.9-77.1)	39.0 (38.9-39.1)	46.2 (46.1-46.3)
**Congestive Heart Failure**					
No	5,387,941 (95.7)	64.9 (64.9-65.0)	67.9 (67.8-67.9)	29.7 (29.7-29.8)	36.2 (36.2-36.3)
Yes	244,472 (4.3)	76.1 (76.0-76.4)	79.6 (79.4-79.7)	42.8 (42.5-43.0)	49.1 (48.9-49.3)
**Diabetes**					
No	4,111,824 (73.0)	61.8 (61.8-61.9)	64.6 (64.6-64.7)	26.7 (26.6-26.7)	32.7 (32.6-32.7)
Yes	1,520,589 (27.0)	75.2 (75.2-75.3)	78.6 (78.6-78.7)	40.1 (40.1-40.2)	48.0 (47.9-48.1)
**Obstructive Sleep Apnea**					
No	4,327,550 (76.8)	63.6 (63.6-63.7)	67.0 (66.9-67.0)	28.7 (28.7-28.8)	35.8 (35.7-35.8)
Yes	1,304,863 (23.2)	71.3 (71.2-71.4)	73.0 (72.9-73.0)	35.2 (35.1-35.3)	39.7 (39.7-39.8)
**Peripheral Arterial Disease**					
No	4,492,979 (79.8)	64.7 (64.6-64.7)	67.6 (67.6-67.7)	29.4 (29.4-29.5)	35.9 (35.8-35.9)
Yes	1,139,434 (20.2)	76.3 (76.1-76.4)	79.7 (79.6-79.9)	42.8 (42.7-43.0)	49.9 (49.7-50.1)
**Venous Thromboembolism**					
No	5,543,831	65.2 (65.2-65.3)	68.2 (68.2-68.3)	30.1 (30.0-30.1)	36.6 (36.5-36.6)

© 2023 Bajema KL et al. *JAMA Network Open.*

	(98.4)				
Yes	88,582 (1.6)	73.2 (72.9-73.5)	75.8 (75.5-76.1)	40.0 (39.7-40.4)	45.6 (45.3-45.9)
**Bipolar Disorder or Schizophrenia**					
No	5,393,119 (95.8)	65.5 (65.5-65.5)	68.5 (68.5-68.5)	30.4 (30.3-30.4)	37.0 (37.0-37.0)
Yes	239,294 (4.6)	63.3 (63.1-63.5)	65.4 (65.2-65.6)	27.1 (26.9-27.3)	30.2 (30.0-30.3)
**Major Depressive Disorder**					
No	4,224,815 (75.0)	65.7 (65.6-65.7)	69.0 (69.0-69.1)	30.9 (30.8-30.9)	38.3 (38.3-38.4)
Yes	1,407,598 (25.0)	64.6 (64.5-64.6)	66.3 (66.2-66.4)	28.3 (28.2-28.4)	31.8 (31.8-31.9)
**Post-Traumatic Stress Disorder**					
No	4,492,979 (79.8)	66.1 (66.0-66.1)	69.5 (69.4-69.5)	31.0 (31.0-31.1)	38.4 (38.3-38.4)
Yes	1,139,434 (20.2)	62.8 (62.7-62.8)	64.0 (63.9-64.1)	27.2 (27.1-27.3)	30.3 (30.2-30.4)
**Number of primary care visits in prior 2 years**					
1-5	2,237,618 (39.7)	56.8 (56.7-56.9)	61.4 (61.3-61.5)	22.3 (22.2-22.3)	31.1 (31.0-31.1)
6-11	1,645,868 (29.2)	67.9 (67.8-68.0)	70.2 (70.1-70.2)	31.7 (31.6-31.7)	37.5 (37.5-37.6)
≥12	1,748,927 (31.1)	74.1 (74.1-74.2)	75.6 (75.6-75.7)	39.3 (39.2-39.4)	43.3 (43.2-43.4)
**Number of mental health visits in prior 2 years**					
0	3,686,303 (65.5)	66.4 (66.3-66.4)	70.1 (70.1-70.2)	31.5 (31.5-31.6)	39.8 (39.8-39.9)
1-6	971,390	61.6 (61.5-61.7)	63.5 (63.4-63.6)	26.5 (26.4-26.6)	30.0 (29.9-30.1)

© 2023 Bajema KL et al. *JAMA Network Open.*

	(17.3)				
7-19	588,785 (10.5)	65.1 (65.0-65.2)	66.4 (66.3-66.5)	28.9 (28.7-29.0)	31.8 (31.7-32.0)
≥20	385,935 (6.9)	66.0 (65.9-66.2)	67.0 (66.9-67.2)	29.7 (29.5-29.8)	31.7 (31.5-31.8)
**Number of specialty care visits in prior 2 years**					
0	292,113 (5.2)	45.5 (45.3-45.6)	51.4 (51.2-51.6)	15.0 (14.9-15.1)	23.4 (23.2-23.6)
1-5	2,447,383 (43.5)	58.6 (58.5-58.6)	62.6 (62.5-62.6)	22.9 (22.9-23.0)	31.1 (31.0-31.1)
6-10	1,249,089 (22.2)	68.6 (68.5-68.6)	70.8 (70.7-70.8)	31.7 (31.6-31.8)	37.5 (37.4-37.5)
11-20	1,017,444 (18.1)	74.3 (74.3-74.4)	76.0 (75.9-76.1)	39.1 (39.0-39.2)	43.7 (43.6-43.8)
≥21	626,384 (11.1)	80.8 (80.7-80.9)	82.1 (82.0-82.2)	49.3 (49.1-49.4)	52.9 (52.7-53.0)

Abbreviations: CMS, Centers for Medicare and Medicaid Services; EHR, Electronic Health Record; VA, Veterans Affairs; VISN, Veterans Integrated Services Network
[a]Veterans aged 18 years or older with an inpatient, outpatient, or telehealth encounter including a primary care visit in the VA health care system in the 24 months preceding cohort entry on December 1, 2020.
[b]Includes VA EHR and VA Community Care program data
[c]Cumulative incidence among entire cohort
[d]Midwest: VISNs 10,12,15,23; Northeast: VISNs 1,2,4-5; South: VISNs 6-9, 16-17; West: VISNs 19-22

© 2023 Bajema KL et al. *JAMA Network Open.*

eFigure. Cumulative Incidence of COVID-19 Primary Vaccination (A-B), First Booster (C-D), and Second Booster Vaccination (E-F) Among U.S. Veterans by Number of Primary and Specialty Care Visits in the Prior 2 Years, December 1, 2020-June 30, 2022

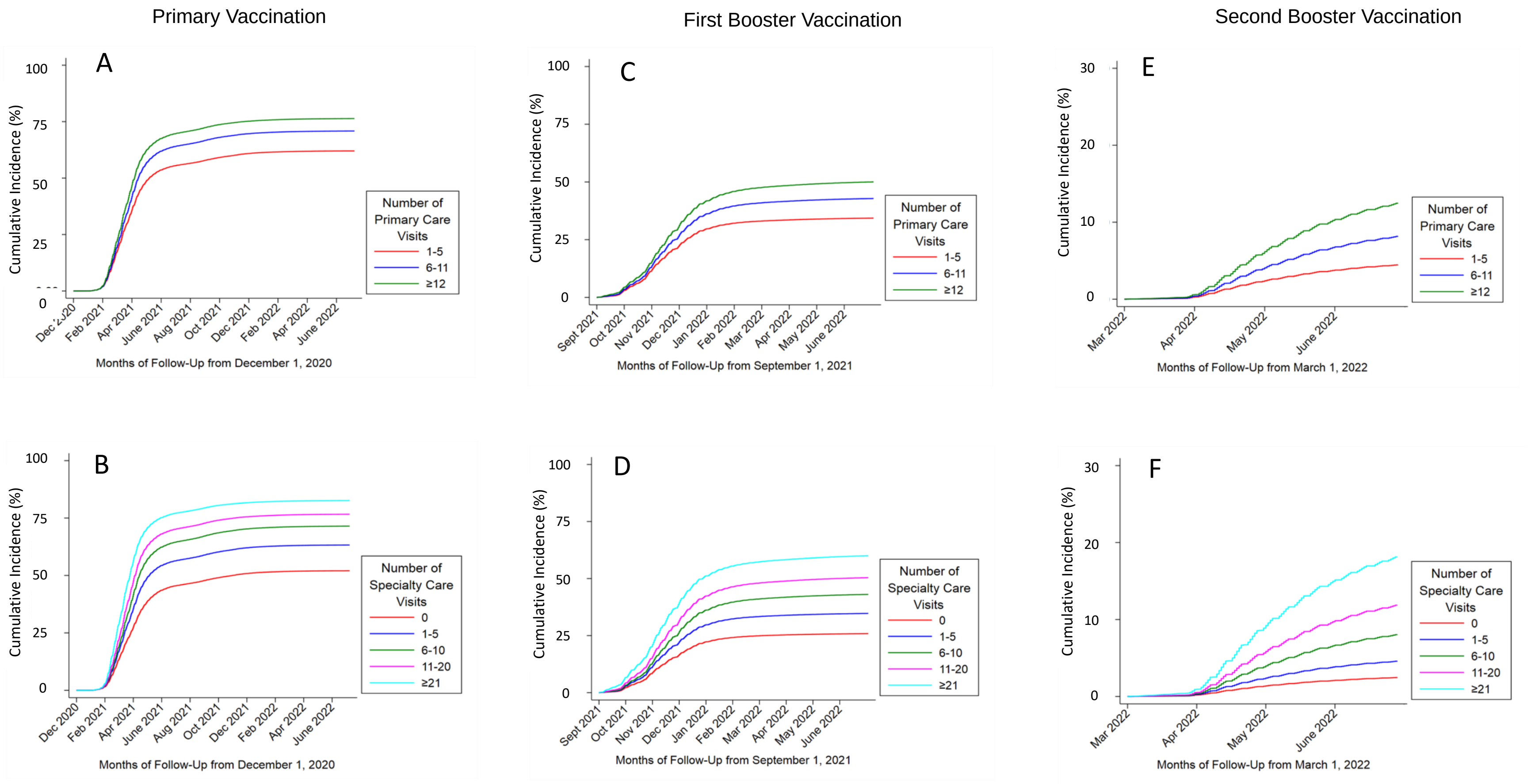

© 2023 Bajema KL et al. *JAMA Network Open.*
