# Supplementary material for: Rates of and Factors Associated With Primary and Booster COVID-19 Vaccine Receipt by US Veterans, December 2020 to June 2022
Source: JAMA Netw Open. 2023 Feb 2;6(2):e2254387. doi: 10.1001/jamanetworkopen.2022.54387 (PMC9896301; doi:10.1001/jamanetworkopen.2022.54387)
Supplement: Supplement 2. — Data Sharing Statement [file jamanetwopen-e2254387-s002.pdf]

Data Sharing Statement

Bajema. Rates of and Factors Associated With Primary and Booster COVID-19 Vaccine Receipt by US Veterans, December 2020 to June 2022. *JAMA Netw Open*. Published February 02, 2023. doi:10.1001/jamanetworkopen.2022.54387

Data
Data available: No
